# Supplementary material for: Modeling Liver Development and Disease in a Dish
Source: Int J Mol Sci. 2023 Nov 2;24(21):15921. doi: 10.3390/ijms242115921 (PMC10650907; doi:10.3390/ijms242115921)
Supplement: Supplementary file 1 [file ijms-24-15921-s001.zip › ijms-2633794-supplementary.pdf]

Table S1.

Organ	NCT Number	Study Title	Link
**Liver**	NCT05183425	Patient-derived Organoids Predicts the Clinical Efficiency of Colorectal Liver Metastasis	https://clinicaltrials.gov/study/NCT05183425?term=NCT05183425&rank=1
**Pancreas**	NCT04777604	Development of a Prediction Platform for Neoadjuvant Treatment and Prognosis in Pancreatic Cancer Using Organoid	https://clinicaltrials.gov/study/NCT04777604?term=NCT04777604&rank=1
	NCT04736043	Development of a Prediction Platform for Adjuvant Treatment and Prognosis in Resected Pancreatic Cancer Using Organoid	https://clinicaltrials.gov/study/NCT04736043?term=NCT04736043&rank=1
	NCT05351983	Patient-derived Organoids Drug Screen in Pancreatic Cancer	https://clinicaltrials.gov/study/NCT05351983?term=NCT05351983&rank=1
	NCT04931381	Organoid-Guided Chemotherapy for Advanced Pancreatic Cancer	https://clinicaltrials.gov/study/NCT04931381?term=NCT04931381&rank=1
	NCT04931394	Organoid-Guided Adjuvant Chemotherapy for Pancreatic Cancer	https://clinicaltrials.gov/study/NCT04931394?term=NCT04931394&rank=1
	NCT05842187	In Vitro Organoid Drug Sensitivity-Guided Treatment for Metastatic Pancreatic and Gastric Cancer	https://clinicaltrials.gov/study/NCT05842187?term=NCT05842187&rank=1
**Brain**	NCT05772741	Grafts of GSCs Into Brain Organoids for Testing Anti-invasion Drugs	https://clinicaltrials.gov/study/NCT05772741?term=NCT05772741&rank=1
**Kidney**	NCT04342286	To Establish a Reproducible Organoid Culture Model with Human Kidney Cancer	https://clinicaltrials.gov/study/NCT04342286?term=NCT04342286&rank=1
**Intestine**	NCT04497727	Gut Organoid Study	https://clinicaltrials.gov/study/NCT04497727?term=NCT04497727&rank=1
	NCT03256266	Effect of Antigens or Therapeutic Agents on in Vitro Human Intestinal Organoids	https://clinicaltrials.gov/study/NCT03256266?term=NCT03256266&rank=1
	NCT05294107	Intestinal Organoids	https://clinicaltrials.gov/study/NCT05294107?term=NCT05294107&rank=1
	NCT02874365	Intestinal Stem Cells Characterization	https://clinicaltrials.gov/study/NCT02874365?term=NCT02874365&rank=1
	NCT05832398	Precision Chemotherapy Based on Organoid Drug Sensitivity for Colorectal Cancer	https://clinicaltrials.gov/study/NCT05832398?term=NCT05832398&rank=1
	NCT05425901	Preclinical Evaluation of Multimodal Therapeutic	https://clinicaltrials.gov/study/NCT05425901?term=NCT05425901&rank=1

		Strategies in Intestinal Irradiation and Inflammatory Bowel Disease from Organoids	
	NCT04371198	Patient-Derived Organoids for Rectal Cancer	https://clinicaltrials.gov/study/NCT04371198?term=NCT04371198&rank=1
	NCT04996355	Organoids-on-a-chip for Colorectal Cancer and in Vitro Screening of Chemotherapeutic Drugs	https://clinicaltrials.gov/study/NCT04996355?term=NCT04996355&rank=1
	NCT05352165	The Clinical Efficacy of Drug Sensitive Neoadjuvant Chemotherapy Based on Organoid Versus Traditional Neoadjuvant Chemotherapy in Advanced Rectal Cancer	https://clinicaltrials.gov/study/NCT05352165?term=NCT05352165&rank=1
	NCT05183425	Patient-derived Organoids Predicts the Clinical Efficiency of Colorectal Liver Metastasis	https://clinicaltrials.gov/study/NCT05183425?term=NCT05183425&rank=1
	NCT05304741	The Culture of Advanced/Recurrent/Metastatic Colorectal Cancer Organoids and Drug Screening	https://clinicaltrials.gov/study/NCT05304741?term=NCT05304741&rank=1
	NCT04906733	Cetuximab Sensitivity Correlation Between Patient-Derived Organoids and Clinical Response in Colon Cancer Patients.	https://clinicaltrials.gov/study/NCT04906733?term=NCT04906733&rank=1
**Lungs**	NCT04859166	Prospective Primary Human Lung cancer Organoids to Predict Treatment Response	https://clinicaltrials.gov/study/NCT04859166?term=NCT04859166&rank=1
	NCT05669586	Organoids Predict Therapeutic Response in Patients with Multi-line Drug-resistant Non-small Cell Lung Cancer	https://clinicaltrials.gov/study/NCT05669586?term=NCT05669586&rank=1
	NCT03655015	Patient-derived Organoid Model and Circulating Tumor Cells for Treatment Response of Lung Cancer	https://clinicaltrials.gov/study/NCT03655015?term=NCT03655015&rank=1
	NCT05092009	Lung Cancer Organoids and Patient Derived Tumor Xenografts	https://clinicaltrials.gov/study/NCT05092009?term=NCT05092009&rank=1
	NCT03979170	Patient-derived Organoids of Lung Cancer to Test Drug Response	https://clinicaltrials.gov/study/NCT03979170?term=NCT03979170&rank=1
	NCT05136014	Evaluation of the Response to Tyrosine Kinase Inhibitors in Localized Non-small Cell Lung Cancer (NSCLC) Patients with EGFR Mutation in a Patient-derived Organoid Model	https://clinicaltrials.gov/study/NCT05136014?term=NCT05136014&rank=1

Gastric	NCT05842187	In Vitro Organoid Drug Sensitivity-Guided Treatment for Metastatic Pancreatic and Gastric Cancer	https://clinicaltrials.gov/study/NCT05842187?term=NCT05842187&rank=1
	NCT05652348	Response Prediction of Hyperthermic Intraperitoneal Chemotherapy in Gastro-Intestinal Cancer	https://clinicaltrials.gov/study/NCT05652348?term=NCT05652348&rank=1
	NCT05351398	The Clinical Efficacy of Drug Sensitive Neoadjuvant Chemotherapy Based on Organoid Versus Traditional Neoadjuvant Chemotherapy in Advanced Gastric Cancer	https://clinicaltrials.gov/study/NCT05351398?term=NCT05351398&rank=1
	NCT05203549	Consistency Between Treatment Responses in PDO Models and Clinical Outcomes in Gastric Cancer	https://clinicaltrials.gov/study/NCT05203549?term=NCT05203549&rank=1
